# Estimating protection afforded by prior infection in preventing reinfection: applying the test-negative study design

**DOI:** 10.1093/aje/kwad239

**Published:** 2023-12-07

**Authors:** Houssein H Ayoub, Milan Tomy, Hiam Chemaitelly, Heba N Altarawneh, Peter Coyle, Patrick Tang, Mohammad R Hasan, Zaina Al Kanaani, Einas Al Kuwari, Adeel A Butt, Andrew Jeremijenko, Anvar Hassan Kaleeckal, Ali Nizar Latif, Riyazuddin Mohammad Shaik, Gheyath K Nasrallah, Fatiha M Benslimane, Hebah A Al Khatib, Hadi M Yassine, Mohamed G Al Kuwari, Hamad Eid Al Romaihi, Hanan F Abdul-Rahim, Mohamed H Al-Thani, Abdullatif Al Khal, Roberto Bertollini, Laith J Abu-Raddad

**Affiliations:** Mathematics Program, Department of Mathematics and Statistics, College of Arts and Sciences, Qatar University, Doha, Qatar; Mathematics Program, Department of Mathematics and Statistics, College of Arts and Sciences, Qatar University, Doha, Qatar; Infectious Disease Epidemiology Group, Weill Cornell Medicine–Qatar, Cornell University, Doha, Qatar; World Health Organization Collaborating Centre for Disease Epidemiology Analytics on HIV/AIDS, Sexually Transmitted Infections, and Viral Hepatitis, Weill Cornell Medicine–Qatar, Cornell University, Qatar Foundation–Education City, Doha, Qatar; Infectious Disease Epidemiology Group, Weill Cornell Medicine–Qatar, Cornell University, Doha, Qatar; World Health Organization Collaborating Centre for Disease Epidemiology Analytics on HIV/AIDS, Sexually Transmitted Infections, and Viral Hepatitis, Weill Cornell Medicine–Qatar, Cornell University, Qatar Foundation–Education City, Doha, Qatar; Department of Population Health Sciences, Weill Cornell Medicine, Cornell University, New York, NY 10065, United States; Infectious Disease Epidemiology Group, Weill Cornell Medicine–Qatar, Cornell University, Doha, Qatar; World Health Organization Collaborating Centre for Disease Epidemiology Analytics on HIV/AIDS, Sexually Transmitted Infections, and Viral Hepatitis, Weill Cornell Medicine–Qatar, Cornell University, Qatar Foundation–Education City, Doha, Qatar; Department of Population Health Sciences, Weill Cornell Medicine, Cornell University, New York, NY 10065, United States; Hamad Medical Corporation, Doha, Qatar; Biomedical Research Center, Member of QU Health, Qatar University, Doha, Qatar; Wellcome-Wolfson Institute for Experimental Medicine, Queens University, Belfast BT9 7BL, United Kingdom; Department of Pathology, Sidra Medicine, Doha, Qatar; Department of Pathology, Sidra Medicine, Doha, Qatar; Hamad Medical Corporation, Doha, Qatar; Hamad Medical Corporation, Doha, Qatar; Department of Population Health Sciences, Weill Cornell Medicine, Cornell University, New York, NY 10065, United States; Hamad Medical Corporation, Doha, Qatar; Hamad Medical Corporation, Doha, Qatar; Hamad Medical Corporation, Doha, Qatar; Hamad Medical Corporation, Doha, Qatar; Hamad Medical Corporation, Doha, Qatar; Biomedical Research Center, Member of QU Health, Qatar University, Doha, Qatar; Department of Biomedical Science, College of Health Sciences, Member of QU Health, Qatar University, Doha, Qatar; Biomedical Research Center, Member of QU Health, Qatar University, Doha, Qatar; Department of Biomedical Science, College of Health Sciences, Member of QU Health, Qatar University, Doha, Qatar; Department of Biomedical Science, College of Health Sciences, Member of QU Health, Qatar University, Doha, Qatar; Biomedical Research Center, Member of QU Health, Qatar University, Doha, Qatar; Department of Biomedical Science, College of Health Sciences, Member of QU Health, Qatar University, Doha, Qatar; Primary Health Care Corporation, Doha, Qatar; Ministry of Public Health, Doha, Qatar; Department of Public Health, College of Health Sciences, Member of QU Health, Qatar University, Doha, Qatar; Ministry of Public Health, Doha, Qatar; Hamad Medical Corporation, Doha, Qatar; Ministry of Public Health, Doha, Qatar; Infectious Disease Epidemiology Group, Weill Cornell Medicine–Qatar, Cornell University, Doha, Qatar; World Health Organization Collaborating Centre for Disease Epidemiology Analytics on HIV/AIDS, Sexually Transmitted Infections, and Viral Hepatitis, Weill Cornell Medicine–Qatar, Cornell University, Qatar Foundation–Education City, Doha, Qatar; Department of Population Health Sciences, Weill Cornell Medicine, Cornell University, New York, NY 10065, United States; Department of Public Health, College of Health Sciences, Member of QU Health, Qatar University, Doha, Qatar

**Keywords:** reinfection, test-negative design, effectiveness, mathematical model, SARS-CoV-2, COVID-19

## Abstract

The COVID-19 pandemic has highlighted the need to use infection testing databases to rapidly estimate effectiveness of prior infection in preventing reinfection ($P{E}_S$) by novel severe acute respiratory syndrome coronavirus 2 (SARS-CoV-2) variants. Mathematical modeling was used to demonstrate a theoretical foundation for applicability of the test-negative, case–control study design to derive $P{E}_S$. Apart from the very early phase of an epidemic, the difference between the test-negative estimate for $P{E}_S$ and true value of $P{E}_S$ was minimal and became negligible as the epidemic progressed. The test-negative design provided robust estimation of $P{E}_S$ and its waning. Assuming that only 25% of prior infections are documented, misclassification of prior infection status underestimated $P{E}_S$, but the underestimate was considerable only when > 50% of the population was ever infected. Misclassification of latent infection, misclassification of current active infection, and scale-up of vaccination all resulted in negligible bias in estimated $P{E}_S$. The test-negative design was applied to national-level testing data in Qatar to estimate $P{E}_S$ for SARS-CoV-2. $P{E}_S$ against SARS-CoV-2 Alpha and Beta variants was estimated at 97.0% (95% CI, 93.6-98.6) and 85.5% (95% CI, 82.4-88.1), respectively. These estimates were validated using a cohort study design. The test-negative design offers a feasible, robust method to estimate protection from prior infection in preventing reinfection.

## Introduction

Estimating effectiveness of prior infection in preventing reinfection ($P{E}_S$) is essential to understanding the epidemiology of a given infection. Various studies estimated $P{E}_S$ for severe acute respiratory syndrome coronavirus 2 (SARS-CoV-2) variants.^1^^-^^9^ However, there are challenges in estimating $P{E}_S$ using conventional epidemiologic study designs. Such designs require extensive, complete electronic health records to be feasible. Vaccination scale-up makes it difficult to disentangle immunity induced by prior infection from that induced by vaccination.

Even when it is feasible to apply conventional designs, estimates can be prone to strong bias, due to misclassification of prior infection status, since many prior infections are not documented.^10^^-^^12^ Effects of this bias increase with increased cumulative infection exposure in the population.^13^ Emergence of the Omicron^14^ (B.1.1.529) variant and its subsequent subvariants emphasized the need to estimate $P{E}_S$ rapidly once a new variant/subvariant emerges.

Here, we demonstrate a robust, practical method to estimate $P{E}_S$ using a test-negative, case–control study design. This is, to our knowledge, the first use of this method to estimate $P{E}_S$. While it has been used to study vaccine effectiveness,^15^^-^^22^ it does not appear to have been used to estimate $P{E}_S$, perhaps because of a perception that it is not applicable, as most prior and current infections are undocumented, unlike vaccinations, which are typically documented and tracked in health systems. We also provide an application of this method by estimating $P{E}_S$ for SARS-CoV-2 infection in Qatar, at a time when the Alpha^14^ (B.1.1.7) and Beta^14^ (B.1.351) variants dominated incidence.^21^^-^^26^

This article includes two components. The first is a parsimonious mathematical modeling component whose purpose is to motivate the test-negative design and to demonstrate that theoretically it can be applied to provide credible estimates for $P{E}_S$ despite specific sources of bias. This modeling exercise is not intended to provide a simulation of a specific empirical study or discuss all sources of potential bias, but to provide a theoretical foundation of the applicability of such design to estimate $P{E}_S$. The second component is a real-world application of the test-negative design to actual routine data to generate estimates for $P{E}_S$. This specific application was conducted because there are already published estimates for $P{E}_S$ using a cohort study design applied to the same data, population, and duration of study.^4^ Therefore, the cohort study design provides a validation for the test-negative design, as both the cohort and test-negative designs yielded the same results when applied to the same data source.

## Methods

### Test-negative case–control study design

The test-negative, case–control study design has emerged as a robust and practical method to assess vaccine effectiveness for respiratory tract infections.^15^^-^^22^^,^^27^^-^^32^ In this design, which resembles a case–control design although it is not strictly a case–control design, persons seeking health care because of symptoms are recruited into the study.^15^^,^^16^^,^^27^^,^^28^^,^^30^^-^^32^ Those testing positive for the infection (cases) are then matched to those testing negative (controls).^15^^,^^16^^,^^27^^,^^28^^,^^30^^-^^32^ Matching is done to control for differences in the risk of exposure to the infection.^21^^,^^22^^,^^33^ Vaccine effectiveness is then derived as 1 minus the ratio of the odds of vaccination in subjects testing positive to the odds of vaccination in subjects testing negative.^15^^,^^16^ A key strength of this design is removal of differences in health care–seeking behavior between vaccinated and unvaccinated persons, thereby minimizing bias.^15^^,^^16^^,^^27^^-^^32^ Another strength is minimization of bias arising from misclassification of infection.^15^^,^^16^^,^^27^^-^^32^

### Mathematical modeling and simulation of the test-negative design

Mathematical modeling was used to demonstrate a theoretical foundation for the applicability of the test-negative, case–control study design for deriving effectiveness of prior infection in preventing reinfection ($P{E}_S$), that is, the proportional reduction in susceptibility to infection among those with prior infection versus those without.^2^ Modeling was also used to investigate effects of biases on estimated $P{E}_S$. While this demonstration was done for SARS-CoV-2 infection, the approach is generic and should be broadly applicable to a range of infections. Moreover, while this demonstration was done for any SARS-CoV-2 infection, regardless of symptoms, the same approach can be applied to other outcomes such as symptomatic infection, asymptomatic infection, severe or critical COVID-19,^34^ or COVID-19 death,^35^ as long as these outcomes are defined as specific subsets of the broad any-infection outcome or its direct disease progression.

Several models were devised to simulate SARS-CoV-2 infection transmission in the population and to investigate applicability of the test-negative design. The models were based on previously published models and their parameters for SARS-CoV-2 infection.^12^^,^^36^^-^^42^ To keep only the essential details for the investigations of this study, the models were parsimonious and not structured by age, nor by infection type and severity. The instantaneous prevalence at each time point, for each population compartment, was used in the analyses of these models.

**Figure 1:**
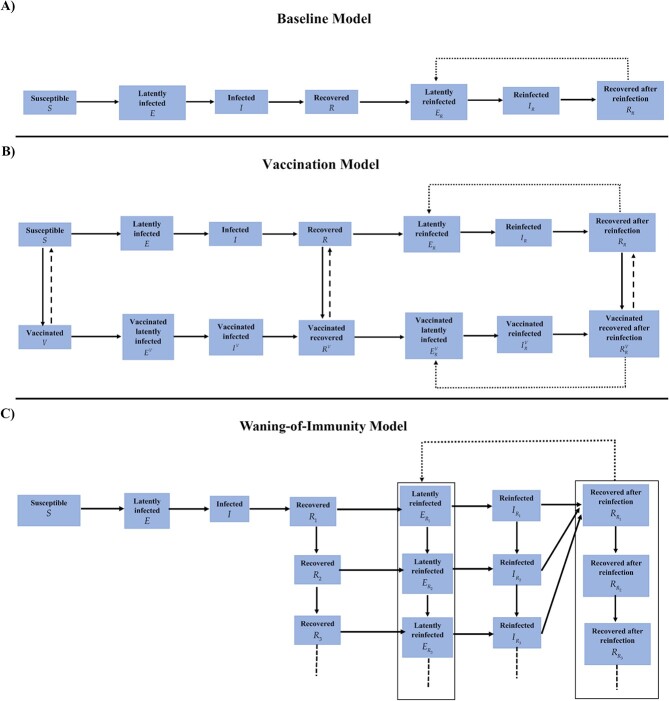
Schematic diagrams of mathematical models used in this study. A) Classic susceptible-exposed-infectious-recovered (SEIR) model extended to allow for reinfections (baseline model). B) Baseline model extended to include vaccination (vaccination model). C) Baseline model extended to include waning in protection of prior infection against reinfection (waning-of-immunity model).

The first model was the classic susceptible-exposed-infectious-recovered (SEIR) model, but extended to allow for reinfections (baseline model; Figure 1A). This model was used to demonstrate applicability of the test-negative design and to investigate sources of bias. In this model and its analysis all controls were either susceptible or recovered individuals, and all cases were either infected or reinfected individuals.

Building on previous modeling studies of vaccine effectiveness and its waning,^13^^,^^43^^-^^47^ the second model was an extension of the baseline model to incorporate scale-up of vaccination in the population (vaccination model; Figure 1B). This model was used to investigate whether vaccination could affect applicability of this method to estimate $P{E}_S$. Vaccine effectiveness ($V{E}_S$) was defined as the proportional reduction in susceptibility to infection among those vaccinated versus those unvaccinated.^40^^,^^41^$V{E}_S$ was set at 75%, a representative value for the range of coronavirus disease 2019 (COVID-19) vaccines available during times in which incidence was due to pre-Omicron variants.^21^^,^^33^^,^^48^^,^^49^ Duration of vaccine-induced protection was assumed to be 6 months in light of documented waning of COVID-19 vaccine protection.^25^^,^^48^^-^^52^

The third model was also an extension of the baseline model, incorporating gradual (linear) waning in protection offered by prior infection against reinfection (waning-of-immunity model; Figure 1C). Time after recovery from infection was modeled as an aging process whereby the recovered population transitions from one population compartment to the next with the average duration spent in each compartment being one month. Each 1-month recovered-population compartment had a set $P{E}_S$ value. $P{E}_S$ was modeled to decline linearly month by month. Accordingly, the recovered population is tracked month by month after recovery to allow for test-negative-study estimation of waning of natural immunity, as is described in the literature for waning of vaccine immunity after the second or booster doses.^25^^,^^52^^,^^53^

These models consisted of coupled nonlinear differential equations that stratified the population into compartments (groups) based on infection status (infected, reinfected, or uninfected) and vaccination status (vaccinated, unvaccinated). Susceptible individuals (vaccinated or unvaccinated) were assumed at risk of acquiring the infection at a force of infection that varied throughout the epidemic due to variation in the contact rate. Recovered individuals (vaccinated or unvaccinated) were also assumed at risk of acquiring the infection, but the force of infection was reduced by the effect of $P{E}_S$.

These models were calibrated to mimic the actual evolution of the COVID-19 epidemic in Qatar.^12^^,^^36^ The contact rate was varied to generate 2 major epidemic waves several months apart, as actually occurred.^12^^,^^25^^,^^36^^,^^54^ Parameters of the models are summarized in Table 1. Further details on these models, their equations, and their parameterization can be found in previous publications.^12^^,^^36^^-^^42^ Modeling analyses were performed using MATLAB R2019a (Natick, MA).^55^

**Table 1:** Model parameters and assumptions.

**Parameter**	**Symbol**	**Value**	**Justification**
Duration of latent infection		3.69 days	Based on existing estimate^83^ and based on a median incubation period of 5.1 days^84^ adjusted by observed viral load among infected persons^85^ and reported transmission before onset of symptoms^86^
Duration of infectiousness		3.48 days	Based on existing estimate^83^ and based on observed time to recovery among persons with mild infection^83^^,^^87^ and observed viral load in infected persons^85^^,^^86^
Infection fatality rate		1.85 per 100 000 infections	Estimate based on fitting the epidemic in Qatar^38^
Life expectancy in Qatar		80.7 years	United Nations World Population Prospects database^88^
Vaccine effectiveness in reducing susceptibility to infection	$V{E}_S$	75%	Representative value for the range of COVID-19 vaccines available at present^21^^,^^33^^,^^48^^,^^49^
Duration of vaccine protection		6 months	Based on evidence on waning of vaccine protection^25^^,^^48^^-^^52^
Model-assumed “true” effectiveness of prior infection in preventing reinfection	$P{E}_S^{true}$	80%	Informed by evidence from existing studies^1^^-^^9^
Proportion of prior infections that are undocumented	${g}_p$	75%	Informed by evidence from existing studies^10^^-^^12^^,^^38^
Proportion of latent infections that are undocumented	${g}_E$	75%	Informed by evidence from existing studies^10^^-^^12^^,^^38^
Proportion of current active infections that are undocumented	${g}_I$	75%	Informed by evidence from existing studies^10^^-^^12^^,^^38^

### Effectiveness of prior infection against reinfection and impact of bias

**Figure 2:**
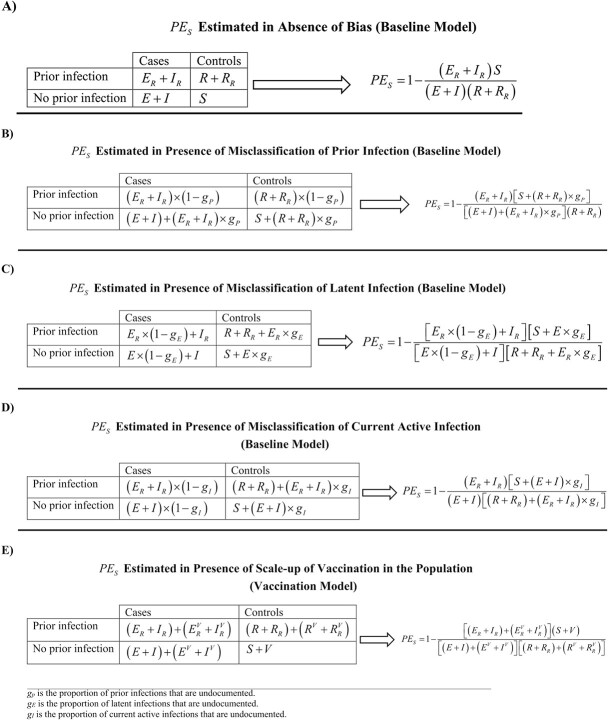
The 2-by-2 tables and equations used to estimate effectiveness of prior infection in preventing reinfection ($P{E}_S$) using the test-negative, case–control study design. A) $P{E}_S$ estimated in absence of bias. B) $P{E}_S$ estimated in presence of misclassification of prior infection. C) $P{E}_S$ estimated in presence of misclassification of latent infection. D) $P{E}_S$ estimated in presence of misclassification of current active infection. E) $P{E}_S$ estimated in presence of vaccination scale-up.

Applying the test-negative, case–control study design, $P{E}_S$ was derived as 1 minus the ratio of the odds of prior infection in subjects testing positive (such as by polymerase chain reaction [PCR] testing) to the odds of prior infection in subjects testing negative for the infection. The 2-by-2 table used to derive the odds ratio is shown in Figure 2A, as expressed in terms of the baseline model’s population variables. The mathematical expression for $P{E}_S$ is also shown in Figure 2A, assuming no form of bias. An underlying assumption is that those being tested are a specific fixed proportion (random sample) of all population variables; that is, the same sampling proportion is applied for each population compartment in the model. We also assumed that those latently infected (*E* compartment) are as diagnosable as those in acute infection (*I* compartment), given the wide application of the highly sensitive PCR testing for SARS-CoV-2 infection, and because of existence of large-scale routine testing in many countries, in addition to testing for symptomatic cases. A departure of the latter assumption has been investigated in a sensitivity analysis.

Several forms of bias may affect estimation of $P{E}_S$ using the test-negative method. The most critical is misclassification of prior infection status. A proportion ${g}_P$ of those previously infected may not have been diagnosed and may have been unaware of their infections. It is reasonable to assume that most persons with a prior infection may not have had it documented.^10^^-^^12^ Here, we assumed that 75% of prior infections are undocumented, that is, an ascertainment rate of only 25% (Table 1). This ascertainment rate was based on fitting epidemic models to national seroprevalence survey data in Qatar,^12^^,^^38^^,^^56^^-^^59^ and is consistent with the ascertainment rate estimated for the United States, also, using serological surveys.^10^

Unlike vaccine effectiveness studies, in which records are typically available to track vaccinations,^15^^-^^22^^,^^33^ most persons with prior infection could be misclassified as persons with no prior infection. Similarly, most currently active infections may not be documented. The 2-by-2 table is thus modified for this bias along with the expression for $P{E}_S$ (Figure 2B). It was assumed that this bias affects both cases and controls similarly, a valid assumption considering that both cases and controls are seeking health care because of symptoms. This assumption is central to the test-negative design strategy.^15^^,^^16^^,^^27^^,^^28^^,^^30^^-^^32^

A second source of bias is misclassification of latent infection status. A proportion ${g}_E$ of those with latent infections are asymptomatic, thereby remaining untested and undiagnosed. These cases would be misclassified as controls. The 2-by-2 table is thus modified to accommodate this bias along with the expression for $P{E}_S$ (Figure 2C). We assumed that ${g}_E=75\%$ (Table 1). We also assumed that this bias similarly affects those with and without prior infection. This is a valid assumption considering that both are seeking health care for the same reason, another assumption central to the test-negative design strategy.^15^^,^^16^^,^^27^^,^^28^^,^^30^^-^^32^

A proportion ${g}_I$ of cases (current active infections) could be misclassified as controls, because of lack of testing or due to imperfect sensitivity of the testing method, thereby introducing bias. The 2-by-2 table is thus modified for this bias along with the expression for $P{E}_S$ (Figure 2D). We assumed that ${g}_I=75\%$ (Table 1). We also assumed that this bias similarly affects those with and without prior infection.^15^^,^^16^^,^^27^^,^^28^^,^^30^^-^^32^

Estimation of $P{E}_S$ may occur at a time when vaccination is being scaled up, as in the current COVID-19 pandemic. This could introduce bias as vaccination is another form of immune protection. Using the vaccination model, the 2-by-2 table is modified in the presence of vaccination along with the expression for $P{E}_S$ (Figure 2E). We assumed that vaccination is being linearly scaled up to reach the vaccine coverage attained in Qatar during the duration of the simulation. We also assumed that protection of natural immunity and of vaccine immunity act independently of each other, as suggested recently for the effect of hybrid immunity.^53^ Accordingly, protection of hybrid immunity of prior infection ($P{E}_S$) and vaccination ($V{E}_S$) combines as a multiplicative protection effect^53^—hybrid immunity of prior infection and vaccination is superior to that of either prior infection or vaccination separately.^53^^,^^54^^,^^60^

Since different forms of bias may act synergistically when present together, the impact of the above biases was also investigated by applying all of them together at the same time.

### Sensitivity analyses

Four sensitivity analyses were conducted. In the first sensitivity analysis, presented analyses were repeated using the real-world, detailed reference mathematical model that was used to describe the epidemic and forecast its progression in Qatar, to inform policy decision-making (the Qatar model; Figure S1).^12^^,^^36^^,^^38^ This model stratified the population into compartments according to age group, infection status (uninfected, infected, reinfected), infection type (asymptomatic/mild, severe, and critical), COVID-19 disease type (severe or critical disease), and vaccination status (vaccinated, unvaccinated). The model was fitted to the national standardized, integrated, and centralized databases of SARS-CoV-2 diagnosed cases, SARS-CoV-2 PCR and antibody testing, COVID-19 hospitalizations, and COVID-19 mortality,^12^ as well as to data of a series of SARS-CoV-2 epidemiologic studies in Qatar.^1^^-^^3^^,^^38^^,^^42^^,^^57^^-^^59^ The model-fitted indicators and the measured indicators and their comparison have been published previously, as well as an array of model projections for different infection and disease outcomes.^12^^,^^36^^-^^41^^,^^61^ Model fitting was used to estimate key epidemiologic indicators including the ascertainment rates among others. Detailed description of the model, its input data, and fitting are available in the references.^12^^,^^36^^,^^38^

The second sensitivity analysis investigated the representativeness of $P{E}_S$ as derived using the test-negative study design of the true $P{E}_S$, over the full spectrum of possible $P{E}_S$ values. The third sensitivity analysis investigated whether the $P{E}_S$ estimate can vary by using incidence instead of instantaneous prevalence in deriving the estimate. The fourth sensitivity analysis investigated the impact on $P{E}_S$ of full misclassification bias of those latently infected. That is, none of those latently infected are being diagnosed; only those in acute infection are being diagnosed.

### Real-world application: effectiveness of prior infection in preventing reinfection in Qatar

To validate the test-negative design, $P{E}_S$ was estimated in Qatar using national-level routine PCR testing data. Databases include all SARS-CoV-2-related data, with no missing information since pandemic onset, such as PCR tests and vaccinations. Only persons being PCR tested for clinical suspicion of infection due to symptoms between March 8 and April 21, 2021, were eligible for inclusion in this analysis. This study duration was chosen because there are existing estimates for $P{E}_S$ during this time but using a conventional, cohort study design.^4^ This allows validation of the estimate generated using the test-negative design.

Prior infection was defined as a PCR-confirmed infection ≥90 days before a new PCR-positive test.^2^^,^^6^ Individuals infected during the 90 days preceding the PCR test were thus excluded. Based on existing evidence^62^^-^^64^ and viral genome sequencing,^3^^,^^21^ a SARS-CoV-2 Alpha variant case was defined as an *S*-gene “target failure” case using the TaqPath COVID-19 Combo Kit (Thermo Fisher Scientific, Waltham, MA^65^) applying the criterion of a PCR cycle threshold (Ct) value ≤ 30 for both the *N* and *ORF1ab* genes but a negative outcome for the *S* gene.^3^^,^^4^^,^^64^ With essentially only Beta and Alpha cases identified between March 8 and April 21, 2021,^21^^-^^26^ a Beta case was proxied as the complement of the Alpha criterion, that is, any case with a Ct value ≤ 30 for the *N*, *ORF1ab*, and *S* genes.

Only the first PCR-positive test during the study was included for each case, and only the first PCR-negative test during the study was included for each control, per established protocol for the test-negative design.^21^^,^^22^^,^^25^^,^^33^ No Beta-positive cases were included as Alpha-negative controls, or vice versa. The negative controls in both the Alpha and Beta analyses were chosen from the same population of those who tested negative during the study. Alpha and Beta cases were exact-matched 1-to-1 to controls (PCR-negative persons) by sex, 10-year age group, nationality, and calendar week of PCR test. Matching of cases and controls was done to control for known differences in the risk of exposure to SARS-CoV-2 infection in Qatar.^12^^,^^42^^,^^57^^-^^59^

This applied test-negative design, including these specific inclusion and exclusion criteria, was developed over a series of studies^17^^,^^21^^,^^22^^,^^25^^,^^52^^,^^66^ to minimize effects of potential bias, such as retesting after a positive test to check for clearance of infection, or to control the effect of repeat testers.^25^ Extensive sensitivity and additional analyses were conducted in these prior studies to investigate effects of different kinds of potential bias in this design, including investigating different adjustments in the analysis, different approaches for matching,^67^ different approaches for factoring prior infection in the analysis, and other different study inclusion and exclusion criteria.^17^^,^^21^^,^^22^^,^^25^^,^^52^^,^^66^ The applied test-negative design is an outcome of these analyses to optimize the design by minimizing different sources of bias in real-world data. The design was also validated using studies that used control groups to test for null effects,^22^^,^^25^^,^^52^^,^^68^^,^^69^ and also validated using cohort study designs applied to the same population and that yielded findings similar to those of the test-negative design.^21^^,^^22^^,^^66^ Further description of Qatar’s databases and methods of analysis can be found in previous publications.^1^^-^^4^^,^^21^^,^^22^^,^^25^^,^^33^^,^^42^^,^^54^

Sociodemographic characteristics of study samples were described using frequency distributions and measures of central tendency. The odds ratio, comparing odds of prior infection among cases versus controls, and its associated 95% confidence interval (CI) were derived using conditional logistic regression, that is, factoring matching in the study design. This analytical approach is done to minimize potential bias due to variation in epidemic phase^15^^,^^70^ and other confounders.^12^^,^^42^^,^^57^^-^^59^^,^^71^^,^^72^$P{E}_S$ and its associated 95% CI were calculated by applying the following equation:

$$ P{E}_S=1-\mathrm{odds}\ \mathrm{ratio}\ \mathrm{of}\ \mathrm{prior}\ \mathrm{infection}\ \mathrm{among}\ \mathrm{cases}\ \mathrm{versus}\ \mathrm{controls} $$

Statistical analyses were performed using STATA/SE, version 17.0.^73^ The study was approved by the Hamad Medical Corporation and Weill Cornell Medicine–Qatar institutional review boards with waiver of informed consent. The study was reported following the Strengthening the Reporting of Observational Studies in Epidemiology (STROBE) guidelines. The STROBE checklist is found in Table S1.

## Results

### Protection of prior infection using the test-negative design and impact of bias

**Figure 3:**
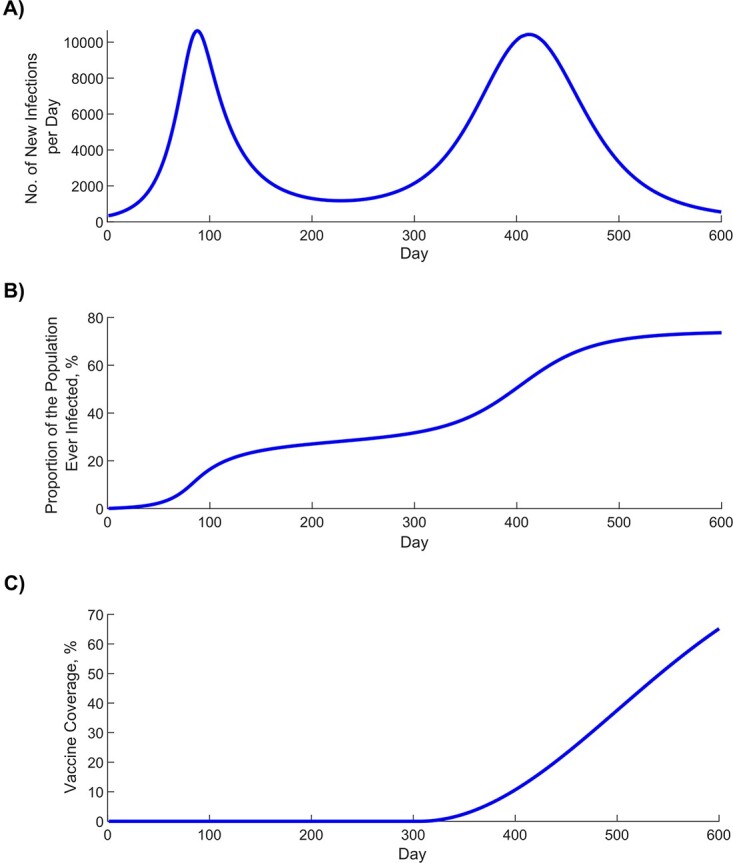
Simulated severe acute respiratory syndrome coronavirus 2 (SARS-CoV-2) epidemic through 2 epidemic waves. A) Daily number of new infections. B) Proportion of the population ever infected. C) Scale-up of vaccine coverage.

**Figure 4:**
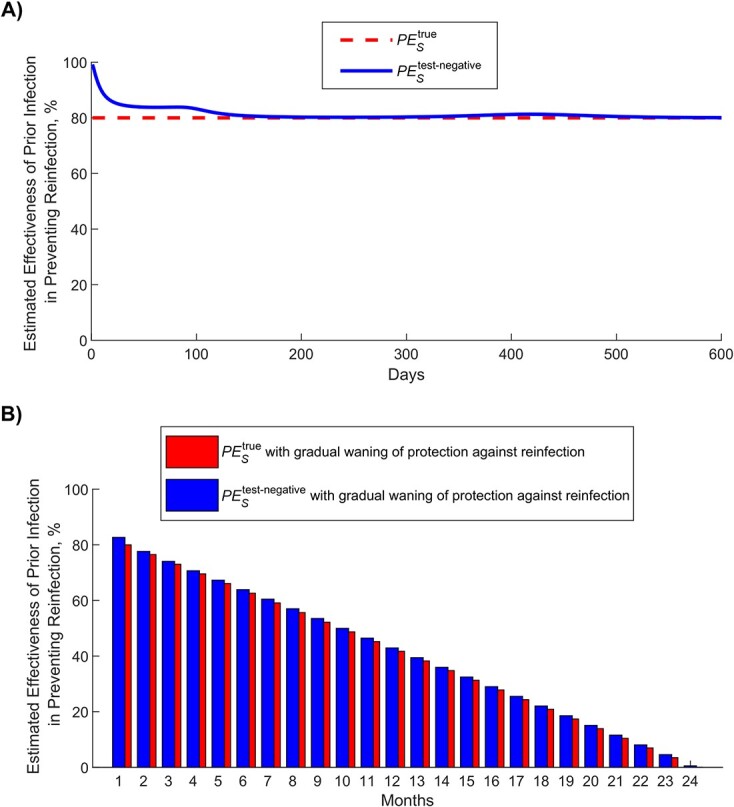
Estimated effectiveness of prior infection in preventing reinfection using the test-negative study design ($P{E}_S^{\mathrm{test}\text{-}\mathrm{negative}}$) compared with the true effectiveness of prior infection in preventing reinfection ($P{E}_S^{\textrm{true}}$). A) $P{E}_S^{\text{test-negative}}$ versus $P{E}_S^{\textrm{true}}$ in presence of no waning of protection (baseline model). B) $P{E}_S^{\text{test-negative}}$ versus $P{E}_S^{\textrm{true}}$ month by month after the prior infection in presence of gradual waning of protection against reinfection (waning-of-immunity model). This figure was generated using the instantaneous prevalence at each time point for each population.

Figure 3 shows simulated evolution of the SARS-CoV-2 epidemic in its 2 waves (Figure 3A), the proportion of the population ever infected (Figure 3B), and vaccine coverage (Figure 3C). Figure 4A shows the estimated $P{E}_S$ using the test-negative design (labeled as $P{E}_S^{\mathrm{test}\text{-}\mathrm{negative}}$), by application of the expression in Figure 2A, compared with the true $P{E}_S$ (labeled as $P{E}_S^{\mathrm{true}}$), here assumed at 80% (Table 1). Apart from the very early phase of the epidemic, when the number of reinfections was minimal, the difference between $P{E}_S^{\mathrm{test}\text{-}\mathrm{negative}}$ and $P{E}_S^{\mathrm{true}}$ was no more than several percentage points. The difference became negligible as the epidemic progressed.

**Figure 5:**
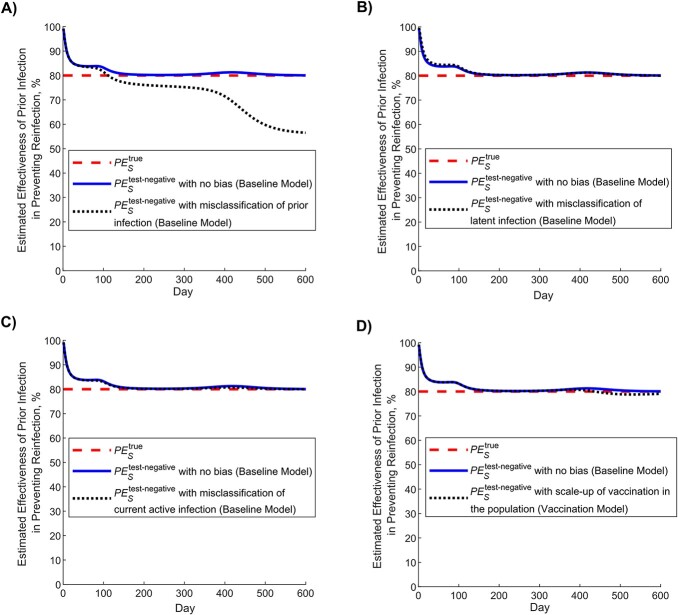
Impact of bias in estimating effectiveness of prior infection in preventing reinfection using the test-negative study design ($P{E}_S^{\mathrm{test}\text{-}\mathrm{negative}}$). A) Impact of misclassification of prior infection. B) Impact of misclassification of latent infection. C) Impact of misclassification of current active infection. D) Impact of scale-up of vaccination in the population. This figure was generated using the instantaneous prevalence at each time point for each population.

Assuming that only 25% of prior infections are documented (Table 1), Figure 5A shows the impact of misclassification of prior infection, by application of the expression in Figure 2B. This form of bias resulted in underestimation of $P{E}_S^{\mathrm{true}}$. When the proportion of the population ever infected was below 50% (Figure 3B), $P{E}_S^{\mathrm{test}\text{-}\mathrm{negative}}$ was only few percentage points lower than that of $P{E}_S^{\mathrm{true}}$. However, the underestimation increased to as much as 30 percentage points when the proportion of the population ever infected was approximately 75%. Therefore, $P{E}_S^{\mathrm{test}\text{-}\mathrm{negative}}$ would provide only a lower bound for $P{E}_S^{\mathrm{true}}$ in situations where nearly everyone is infected, such as for influenza.

Misclassification of latent infection (Figure 5B), misclassification of current active infection (Figure 5C), and scale-up of vaccination (Figure 5D) all resulted in negligible bias in estimated $P{E}_S^{\mathrm{test}\text{-}\mathrm{negative}}$. Application of the above forms of bias at the same time suggested that there is no synergy when biases are combined (Figure S2).

Applying the waning-of-immunity model, Figure 4B shows $P{E}_S^{\mathrm{test}\text{-}\mathrm{negative}}$ versus $P{E}_S^{\mathrm{true}}$, month by month after prior infection, assuming that there is a gradual linear waning in protection of prior infection against reinfection. This comparison was done after the second wave at day 600 after the virus introduction (Figure 3A). $P{E}_S^{\mathrm{test}\text{-}\mathrm{negative}}$ provided a robust approximation of $P{E}_S^{\mathrm{true}}$ and its waning month by month.

Above analyses were repeated in the first sensitivity analysis that used the real-world Qatar model. The analysis confirmed the same findings as those of the main analysis using the parsimonious models (Figure S3). Impact of bias due to scale-up of vaccination was not investigated using the Qatar model, as this model’s fitting already factors in the scale-up of vaccination in Qatar.^36^

The second sensitivity analysis showed that $P{E}_S^{\mathrm{test}\text{-}\mathrm{negative}}$ reflects the value of $P{E}_S^{\mathrm{true}}$ regardless of the actual value of $P{E}_S^{\mathrm{true}}$and over the full spectrum of possible $P{E}_S^{\mathrm{true}}$ values (Figure S4). The third sensitivity analysis showed that the $P{E}_S^{\mathrm{test}\text{-}\mathrm{negative}}$ estimate using incidence is similar to that using instantaneous prevalence (Figure S5). The fourth sensitivity analysis showed that full misclassification bias of those latently infected has virtually no impact on estimated $P{E}_S^{\mathrm{test}\text{-}\mathrm{negative}}$ (Figure S6).

### Application: effectiveness of prior infection in preventing reinfection in Qatar

**Figure 6:**
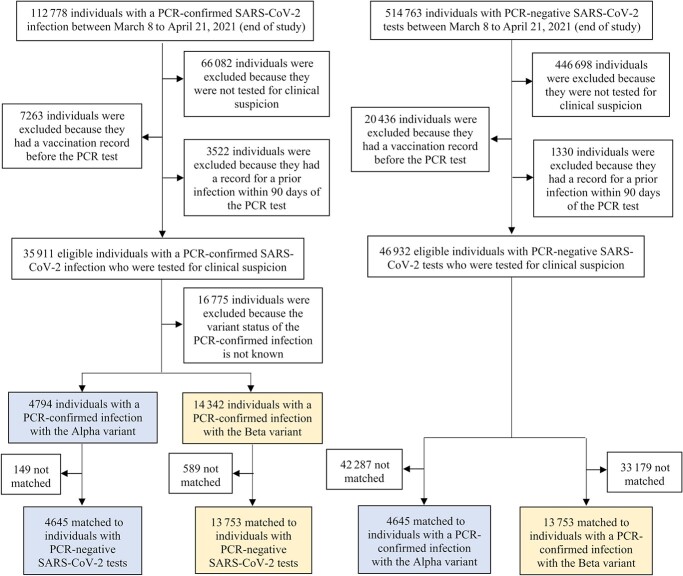
Flowchart describing the population selection process to estimate effectiveness of prior infection in preventing reinfection using the test-negative study design, using data from Qatar, March 8 to April 21, 2021. Individuals with a polymerase chain reaction (PCR)-confirmed infection with severe acute respiratory syndrome coronavirus 2 (SARS-CoV-2) Alpha or Beta variant were exact matched on a 1:1 ratio by sex, 10-year age group, nationality, and PCR test calendar week to the first eligible PCR-negative individual. Prior infection records were retrieved for all matched individuals.

Figure 6 presents a flowchart describing the population selection process for estimating $P{E}_S$ in Qatar using the test-negative design. The median age of study subjects was 32-34 years, at least half were male, and they came from diverse countries (Table 2). Study samples were broadly representative of Qatar’s demographic distributions.^42^^,^^74^

**Table 2:** Demographic characteristics of subjects in the samples used to estimate effectiveness of prior infection in preventing reinfection using the test-negative study design, Qatar, 2021

	**Cases** ^**a**^ **(PCR-confirmed infection with the Alpha variant)**		**Controls** ^**a**^ **(PCR-negative)**			**Cases** ^**a**^ **(PCR-confirmed infection with the Beta variant)**		**Controls** ^**a**^ **(PCR-negative)**		**SMD** ^**b**^
	***n* = 4645**		***n* = 4645**			***n* = 13 753**		***n* = 13 753**		
**Characteristic**	**No.**	**%**	**No.**	**%**	**SMD** ^**b**^	**No.**	**%**	**No.**	**%**	
Age, years	33 (25-40)^c^		32 (24-40)^c^		0.01^d^	34 (27-40)^c^		33 (27-40)^c^		0.01^d^
Age category, years					0.00					0.00
<20 years	868	18.7	868	18.7		1767	12.9	1767	12.9	
20-29 years	923	19.9	923	19.9		2931	21.3	2931	21.3	
30-39 years	1648	35.5	1648	35.5		5213	37.9	5213	37.9	
40-49 years	871	18.8	871	18.8		2877	20.9	2877	20.9	
50-59 years	272	5.9	272	5.9		797	5.8	797	5.8	
60-69 years	53	1.1	53	1.1		132	1.0	132	1.0	
≥70 years	10	0.2	10	0.2		36	0.3	36	0.3	
Sex					0.00					0.00
Male	2339	50.4	2339	50.4		9467	68.8	9467	68.8	
Female	2306	49.6	2306	49.6		4286	31.2	4286	31.2	
Nationality^**e**^					0.00					0.00
Bangladeshi	235	5.1	235	5.1		1334	9.7	1334	9.7	
Egyptian	358	7.7	358	7.7		990	7.2	990	7.2	
Filipino	764	16.5	764	16.5		1610	11.7	1610	11.7	
Indian	789	17.0	789	17.0		3481	25.3	3481	25.3	
Nepalese	170	3.7	170	3.7		1283	9.3	1283	9.3	
Pakistani	192	4.1	192	4.1		542	3.9	542	3.9	
Qatari	762	16.4	762	16.4		1288	9.4	1288	9.4	
Sri Lankan	125	2.7	125	2.7		538	3.9	538	3.9	
Sudanese	166	3.6	166	3.6		442	3.2	442	3.2	
Other nationalities^f^	1084	23.3	1084	23.3		2245	16.3	2245	16.3	
Prior infection^**g**^					0.31					0.28
No prior infection	4638	99.8	4413	95.0		13 629	99.1	12 938	94.1	
>90 days	7	0.2	232	5.0		124	0.9	815	5.9	

Abbreviations: PCR, polymerase chain reaction; SMD, standardized mean difference.

^a^Cases and controls were matched 1-to-1 by sex, 10-year age group, nationality, and calendar week of PCR test.

^b^SMD is the difference in the mean of a covariate between groups divided by the pooled standard deviation. An SMD < 0.1 indicates adequate matching.

^c^Values are expressed as median (interquartile range).

^d^SMD is the mean difference between groups divided by the pooled standard deviation.

^e^Nationalities were chosen to represent the most populous groups in Qatar.

^f^These comprise 61 other nationalities in Qatar in the Alpha variant analysis and 78 other nationalities in the Beta variant analysis.

^g^Given our interest in quantifying differentials in the odds of exposure to prior infection between cases and controls, this variable was not included as a matching factor.

Among the 4645 Alpha cases (PCR-positive persons), 7 had a record of prior infection, compared with 232 among their matched controls (PCR-negative persons). $P{E}_S$ against Alpha was estimated at 97.0% (95% CI, 93.6-98.6). Among the 13 753 Beta cases, 124 had a record of prior infection, compared with 815 among their matched controls. $P{E}_S$ against Beta was estimated at 85.5% (95% CI, 82.4-88.1).

There were 239 discordant pairs and 4406 concordant pairs in the Alpha analysis and 925 discordant pairs and 12 828 concordant pairs in the Beta analysis. The analyses were conducted on large samples of paired cases and controls and should not be affected by bias due to small samples or sparse data.^75^

During the study duration (March 8 to April 21, 2021), we conducted 2 earlier matched cohort studies to estimate $P{E}_S$ for Alpha and for Beta.^4^ For Alpha, cohort-study estimates were 97.6% (95% CI, 95.7-98.7) and 96.4% (95% CI, 92.1-98.3).^4^ For Beta, cohort-study estimates were 92.3% (95% CI, 90.3-93.8) and 86.4% (95% CI, 82.5-89.5).^4^

### Power analysis

The above application for Alpha and Beta protections demonstrates an actual empirical application, but the number of cases may not be sufficient in other applications to provide a precise and meaningful estimate for $P{E}_S$. Therefore, we conducted a power analysis to provide an estimate of the sample size necessary to apply this method using Power and Sample Size, version 3.1.2,^76^ following Dupont principles.^77^

Assuming the proportion of controls with prior infection at 25% and a high correlation between cases and controls of 0.5,^78^ an estimated sample size of 71 individuals for each of cases and controls is needed to detect an odds ratio of 0.2, that is, assuming $P{E}_S$ of 80%, at 2-sided type I error probability of 5% and power of 80%.

Assuming an attrition of 80% due to exclusion for study ineligibility and an additional attrition of 5% from loss to matching, as informed by the above applications for Alpha and Beta protections, the required sample size would be 374 for each cases and controls. If $P{E}_S$ was 50% instead (an odds ratio of 0.5), the required sample size would be 1474 for each of cases and controls.

## Discussion

This study’s results show that the test-negative design can be used to generate rigorous estimates for protection afforded by prior infection against reinfection, even though most prior infections are undocumented. Estimates were robust despite several forms of potential bias, and even under rather extreme assumptions for these biases. The test-negative design was also applied to Qatar’s routine PCR testing data, and results were validated by comparing test-negative estimates with those generated using conventional cohort study designs.^4^ Application of the test-negative design should be feasible in different countries as long as there are databases for infection testing that are of reasonable quality and that can be linked to documented prior infection status (and preferably to vaccination status). Such databases are available and have been used extensively in vaccine effectiveness studies using the test-negative design, such as for SARS-CoV-2 infection,^17^^-^^22^^,^^33^ and recently to estimate $P{E}_S$ for the Omicron variant.^79^ This is a key strength for test-negative studies in that such studies are typically implemented on full eligible routine datasets where the large sample sizes optimize the statistical precision of the estimates.

Of the considered biases, only misclassification of prior infection status could have a large effect on $P{E}_S$ estimation, but mainly where more than 50% of the population already had a prior infection. This situation is not likely to have been reached for SARS-CoV-2 infection before the introduction the Omicron variant in most countries.^56^ Even in such situations, the direction (and magnitude) of bias is known; it underestimates $P{E}_S$. Therefore, the test-negative design can still provide a lower bound for the true $P{E}_S$, which may be sufficient to inform public health decision making, such as in relation to differential application of restrictions according to prior infection status, timing of vaccination following documented infection, and protocols for isolation and quarantine. Thus, this bias may not restrict the utility of this method.

The test-negative study design has strengths that conventional designs may lack. Cohort study designs can be affected by bias resulting from different infection testing frequencies in the different arms of the study. This bias does not affect the test-negative design, as it uses only those who are tested. An example can be seen in comparing the results of the test-negative design with the results of our earlier cohort design.^4^ In the cohort design, adjustment for testing frequency reduced $P{E}_S$ from 97.6% (95% CI, 95.7-98.7) to 95.8% (95% CI, 92.5-97.7) for Alpha,^4^ very similar to the test-negative estimate of 97.0% (95% CI, 93.6-98.6). Similarly for Beta, adjustment for testing frequency reduced $P{E}_S$ from 92.3% (95% CI, 90.3-93.8) to 86.5% (95% CI, 83.0-89.2),^4^ very similar to the test-negative estimate of 85.5% (95% CI, 82.4-88.1). Accordingly, the test-negative design may provide a more representative estimate than the cohort design.

The test-negative design may also be preferable to the cohort design for other reasons. Cohort designs rely on cohorts that may not be strictly comparable, and it may not be possible to control for all differences in risk of exposure to the infection by matching and analysis adjustments. For example, in our earlier cohort study,^4^ we compared those who had a record of a prior PCR-confirmed infection with those who had an antibody-negative test, but these groups may differ in ways that cannot be controlled. Meanwhile, the test-negative design is perhaps less susceptible to such differences, as cases and controls are selected to meet certain clinical criteria that presumably imply the same health care–seeking behavior. That said, use of administrative databases may still be prone to bias due to unmeasured differences in health care–seeking behavior. Last, while the test-negative design can be biased by misclassification of prior infection, the cohort design is perhaps more affected by this bias. The odds ratio metric in the test-negative design is less affected by this bias than the relative risk, incidence rate ratio, or hazard ratio metrics in the cohort design.

With regard to limitations, we used a heuristic approach to motivate the test-negative design through mathematical modeling, but this approach may not exactly match an actual empirical test-negative-design application. The ultimate validity and utility of this design rests on actual validation studies, including comparison with results of other conventional designs. We provided 2 such validation studies in the present study for each of the Alpha and Beta variants. Considering the demonstrated utility of this design in providing timely results in emergent situations during the COVID-19 pandemic,^53^^,^^79^^-^^81^ this study should be seen as a call for further investigation and methodological development to enhance this design and its applications.

Specific forms of bias were investigated, but other sources of bias are possible, and these may also depend on the database being analyzed.^25^ There is already a volume of literature investigating other forms of bias for the test-negative design in the context of vaccine effectiveness estimation,^15^^,^^16^^,^^27^^-^^32^ some of which may also apply in the context of $P{E}_S$ estimation, such as for issues relating to testing and applicability of this design for different testing modalities.^25^ More studies are needed to investigate different methodological aspects of this design and other sources of bias, such as the uncertainty/power to estimate effect and validity of the assumption of proportional random sampling of the different epidemiologic classes/compartments.

While this study demonstrated use of the test-negative design to estimate $P{E}_S$, other factors need to be considered in actual application. For instance, the algorithm for matching^67^^,^^82^ needs to be developed with knowledge of the local epidemiology to ensure that matching can effectively control differences in the risk of exposure to the infection. Of note, with Qatar’s young population, the estimates presented here for $P{E}_S$ may not be generalizable to other countries where elderly citizens constitute a larger proportion of the total population.

The models used to investigate applicability of the test-negative design were not structured by age, nor by infection type and severity. However, the sensitivity analysis that used the real-world Qatar model, with its detailed stratifications, confirmed the same findings as those of the study’s parsimonious models. Moreover, the 3 other sensitivity analyses confirmed the applicability of the test-negative design regardless of the value of $P{E}_S^{\mathrm{true}}$, irrespective of whether incidence is used instead of instantaneous prevalence in the estimation, and whether or not there was full misclassification bias of those latently infected.

In conclusion, the test-negative design offers a feasible and robust method to estimate protection of prior infection in preventing reinfection. This method should be considered to provide rapid, rigorous estimates of protection offered by prior infection for different variants of SARS-CoV-2, including those that emerged recently.

## Supplementary material

Supplementary material is available at *American Journal of Epidemiology* online.

## Acknowledgments

We acknowledge the many dedicated individuals at Hamad Medical Corporation, the Ministry of Public Health, the Primary Health Care Corporation, the Qatar Biobank, Sidra Medicine, and Weill Cornell Medicine—Qatar for their diligent efforts and contributions to make this study possible.

## Funding

H.H.A. acknowledges the support of Qatar University collaborative grant QUCG-CAS-23/24-114 and Marubeni grant M-QJRC-2020-5. The authors are grateful for support from the Biomedical Research Program and the Biostatistics, Epidemiology, and Biomathematics Research Core, at Weill Cornell Medicine–Qatar, as well as for support provided by the Ministry of Public Health, Hamad Medical Corporation, and Sidra Medicine. The authors are also grateful for the Qatar Genome Programme and Qatar University Biomedical Research Center for institutional support for the reagents needed for the viral genome sequencing. The developed mathematical models were made possible thanks to modeling infrastructure developed through National Priorities Research Program (NPRP) grant number 9-040-3-008 (PI: L.J.A.) and NPRP grant number 12S-0216-190094 (PI: L.J.A.) from the Qatar National Research Fund (a member of Qatar Foundation; https://www.qnrf.org). Open Access funding provided by the Qatar National Library.

## Conflict of interest

A.A.B. has received institutional grant funding from Gilead Sciences unrelated to the work presented in this paper. The other authors declare no conflicts of interest.

## Disclaimer

The statements made herein are solely the responsibility of the authors. The funders had no role in study design, data collection and analysis, decision to publish, or preparation of the manuscript.

## Data availability

The dataset of this study is a property of the Qatar Ministry of Public Health that was provided to the researchers through a restricted-access agreement that prevents sharing the dataset with a third party or publicly. The data are available under restricted access for preservation of confidentiality of patient data. Access can be obtained through a direct application for data access to Her Excellency the Minister of Public Health (https://www.moph.gov.qa/english/OurServices/eservices/Pages/Governmental-Health-Communication-Center.aspx). The raw data are protected and are not available due to data privacy laws. Aggregate data are available within the manuscript and its Supplementary Material. The models’ MATLAB codes can be found at the following URL: https://github.com/HousseinAyoub/Estimating-protection-afforded-by-prior-infection-in-preventing-reinfection.git

## Supplementary Material

Web_Material_kwad239
