# Supplementary material for: Estimating protection afforded by prior infection in preventing reinfection: applying the test-negative study design
Source: Am J Epidemiol. 2023 Dec 7;193(6):883–97. doi: 10.1093/aje/kwad239 (PMC11145912; doi:10.1093/aje/kwad239)
Supplement: Web_Material_kwad239 [file web_material_kwad239.zip › kwad239 Ayoub Web Material Final.pdf]

Supplementary Material

Estimating protection afforded by prior infection in preventing reinfection: Applying the test-negative study design

Houssein H. Ayoub, Milan Tomy, Hiam Chemaitelly, Heba N. Altarawneh, Peter Coyle, Patrick Tang, Mohammad R. Hasan, Zaina Al Kanaani, Einas Al Kuwari, Adeel A. Butt Andrew Jeremijenko, Anvar Hassan Kaleeckal, Ali Nizar Latif, Riyazuddin Mohammad Shaik, Gheyath K. Nasrallah Fatiha M. Benslimane Hebah A. Al Khatib Hadi M. Yassine Mohamed G. Al Kuwari, Hamad Eid Al Romaihi, Hanan F. Abdul-Rahim, Mohamed H. Al-Thani, Abdullatif Al Khal, Roberto Bertollini, and Laith J. Abu-Raddad

Table of Contents

Figure S1. Schematic diagram illustrating the structure of the deterministic mathematical model developed to describe SARS-CoV-2 transmission dynamics in the population of Qatar (the Qatar Model). The detailed structure of this model and its description are found in References [1-4]. In this figure, solid lines denote progression or forward movement from one population compartment to the next, while doted/dashed lines denote backward movement from the present population compartment to the previous one. 3

Table S1. STROBE checklist for case-control studies. 4

Figure S2. Impact of combined biases in estimating effectiveness of prior infection in preventing reinfection using the test-negative study design ($PE_S^{test-negative}$). This figure shows the results applying at the same time misclassification of prior infection, misclassification of latent infection, misclassification of current active infection, and scale-up of vaccination in the population. 6

Figure S3. Sensitivity analysis. Impact of bias in estimating effectiveness of prior infection in preventing reinfection using the test-negative study design ($PE_S^{test-negative}$) applied to the data-fitted Qatar Model [1-4]. A) Impact of misclassification of prior infection. B) Impact of misclassification of latent infection. C) Impact of misclassification of current active infection. 7

Figure S4. Sensitivity analysis. Investigation of the representativeness of the effectiveness of prior infection in preventing reinfection using the test-negative study design ($PE_S^{test-negative}$) of the true effectiveness (PE_S^{true}) over the full spectrum of possible PE_S^{true} values. $PE_S^{test-negative}$ is derived here at the infection endemic equilibrium. The figure shows also the linear trend fit of the relationship between PE_S^{true} and $PE_S^{test-negative}$. 8

Figure S5. Sensitivity analysis. Impact of using instantaneous incidence instead of instantaneous prevalence in estimating effectiveness of prior infection in preventing reinfection using the test-negative study design ($PE_S^{test-negative}$). 9

Figure S6. Sensitivity analysis. Impact on estimation of effectiveness of prior infection in preventing reinfection using the test-negative study design ($PE_S^{test-negative}$) if there is full misclassification bias of

those latently infected. Here, none of those latently infected are being diagnosed; only those in acute infection are being diagnosed. 10

References 11

Figure S1. Schematic diagram illustrating the structure of the deterministic mathematical model developed to describe SARS-CoV-2 transmission dynamics in the population of Qatar (the Qatar Model). The detailed structure of this model and its description are found in References [1-4]. In this figure, solid lines denote progression or forward movement from one population compartment to the next, while doted/dashed lines denote backward movement from the present population compartment to the previous one.

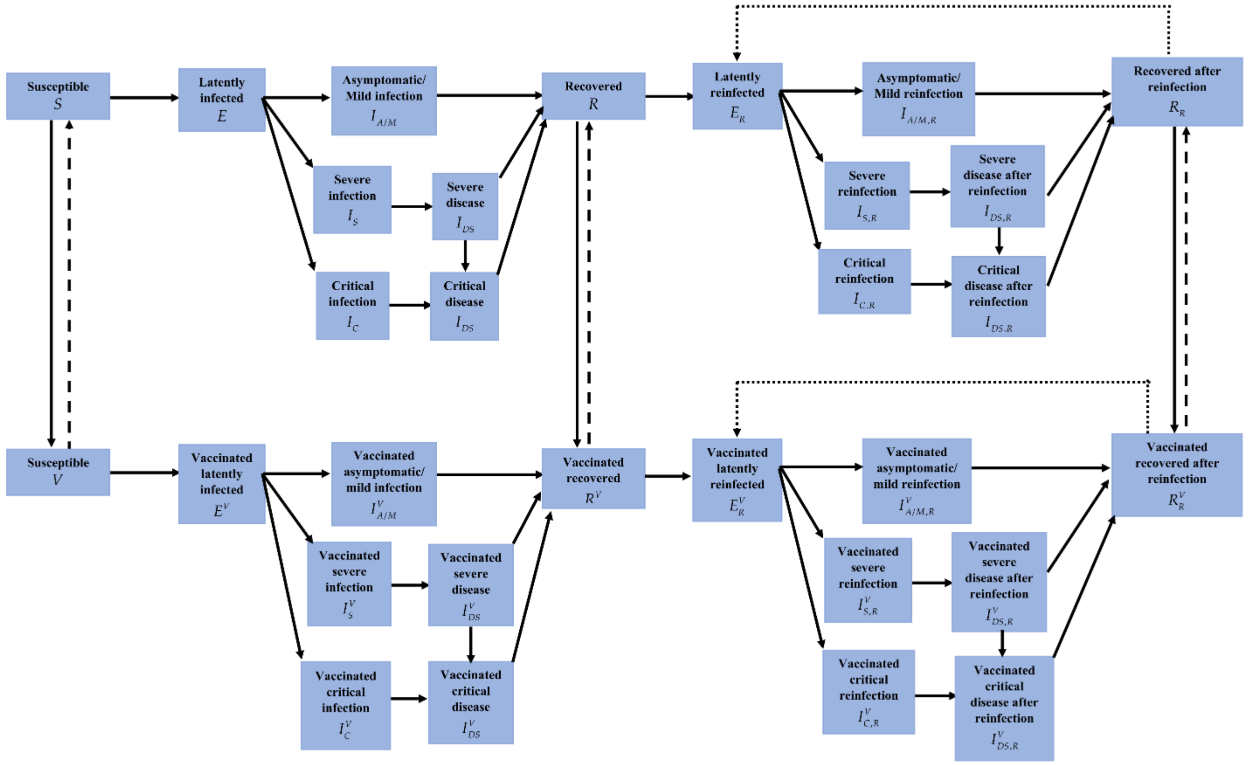

Table S1. STROBE checklist for case-control studies.

	Item No	Recommendation	Main text
**Title and abstract**	1	(*a*) Indicate the study's design with a commonly used term in the title or the abstract	Abstract
		(*b*) Provide in the abstract an informative and balanced summary of what was done and what was found	Abstract
**Introduction**			
Background/rationale	2	Explain the scientific background and rationale for the investigation being reported	Introduction
Objectives	3	State specific objectives, including any prespecified hypotheses	Introduction
**Methods**			
Study design	4	Present key elements of study design	Methods ('Test-negative case-control study design', 'Effectiveness of prior infection against reinfection and impact of bias', & 'Mathematical modeling and simulation of the test-negative design') & Figure 1
Setting	5	Describe the setting, locations, and relevant dates, including periods of recruitment, exposure, follow-up, and data collection	Methods ('Real-world application: Effectiveness of prior infection in preventing reinfection in Qatar')
Participants	6	(*a*) Give the eligibility criteria, and the sources and methods of case ascertainment and control selection. Give the rationale for the choice of cases and controls	Methods ('Real-world application: Effectiveness of prior infection in preventing reinfection in Qatar')
		(*b*) For matched studies, give matching criteria and the number of controls per case	
Variables	7	Clearly define all outcomes, exposures, predictors, potential confounders, and effect modifiers. Give diagnostic criteria, if applicable	Methods ('Test-negative case-control study design', 'Mathematical modeling and simulation of the test-negative design', & 'Real-world application: Effectiveness of prior infection in preventing reinfection in Qatar') & Table 1
Data sources/ measurement	8	For each variable of interest, give sources of data and details of methods of assessment (measurement). Describe comparability of assessment methods if there is more than one group	Methods ('Mathematical modeling and simulation of the test-negative design' & 'Real-world application: Effectiveness of prior infection in preventing reinfection in Qatar') & Table 1
Bias	9	Describe any efforts to address potential sources of bias	Methods ('Mathematical modeling and simulation of the test-negative design', 'Effectiveness of prior infection against reinfection and impact of bias' & 'Real-world application: Effectiveness of prior infection in preventing reinfection in Qatar', paragraph 3)
Study size	10	Explain how the study size was arrived at	Figure 6
Quantitative variables	11	Explain how quantitative variables were handled in the analyses. If applicable, describe which groupings were chosen and why	Figure 2, Tables 1 & 2
Statistical methods	12	(*a*) Describe all statistical methods, including those used to control for confounding	Methods ('Mathematical modeling and simulation of the test-negative design', 'Effectiveness of prior infection against reinfection and impact of bias', 'Sensitivity analyses', & 'Real-world application: Effectiveness of prior infection in preventing reinfection in Qatar')
		(*b*) Describe any methods used to examine subgroups and interactions	Methods ('Effectiveness of prior infection against reinfection and impact of bias' & 'Sensitivity analyses')
		(*c*) Explain how missing data were addressed	Not applicable, see Methods ('Real-world application: Effectiveness of prior infection in preventing reinfection in Qatar')
		(*d*) If applicable, explain how matching of cases and controls was addressed	Methods ('Real-world application: Effectiveness of prior infection in preventing reinfection in Qatar', paragraph 3)

		(*e*) Describe any sensitivity analyses	Methods ('Sensitivity analyses')
**Results**			
Participants	13	(a) Report numbers of individuals at each stage of study—eg numbers potentially eligible, examined for eligibility, confirmed eligible, included in the study, completing follow-up, and analysed	Figure 6
		(b) Give reasons for non-participation at each stage	
		(c) Consider use of a flow diagram	
Descriptive data	14	(a) Give characteristics of study participants (eg demographic, clinical, social) and information on exposures and potential confounders	Table 2
		(b) Indicate number of participants with missing data for each variable of interest	Not applicable, see Methods ('Real-world application: Effectiveness of prior infection in preventing reinfection in Qatar')
Outcome data	15	Report numbers in each exposure category, or summary measures of exposure	Results, & Table 1
Main results	16	(*a*) Give unadjusted estimates and, if applicable, confounder-adjusted estimates and their precision (eg, 95% confidence interval). Make clear which confounders were adjusted for and why they were included	Results, Figures 4 & 5
		(*b*) Report category boundaries when continuous variables were categorized	Tables 1 & 2
		(*c*) If relevant, consider translating estimates of relative risk into absolute risk for a meaningful time period	Not applicable
Other analyses	17	Report other analyses done—eg analyses of subgroups and interactions, and sensitivity analyses	Results, & Figure 5, & Web Figures 2-6 in Supplementary Material
**Discussion**			
Key results	18	Summarise key results with reference to study objectives	Discussion, paragraphs 1-4
Limitations	19	Discuss limitations of the study, taking into account sources of potential bias or imprecision. Discuss both direction and magnitude of any potential bias	Discussion, paragraphs 5-8
Interpretation	20	Give a cautious overall interpretation of results considering objectives, limitations, multiplicity of analyses, results from similar studies, and other relevant evidence	Discussion, paragraph 9
Generalisability	21	Discuss the generalisability (external validity) of the study results	Discussion, paragraphs 5-6
**Other information**			
Funding	22	Give the source of funding and the role of the funders for the present study and, if applicable, for the original study on which the present article is based	Acknowledgement

Figure S2. Impact of combined biases in estimating effectiveness of prior infection in preventing reinfection using the test-negative study design ($PE_S^{test-negative}$). This figure shows the results applying at the same time misclassification of prior infection, misclassification of latent infection, misclassification of current active infection, and scale-up of vaccination in the population.

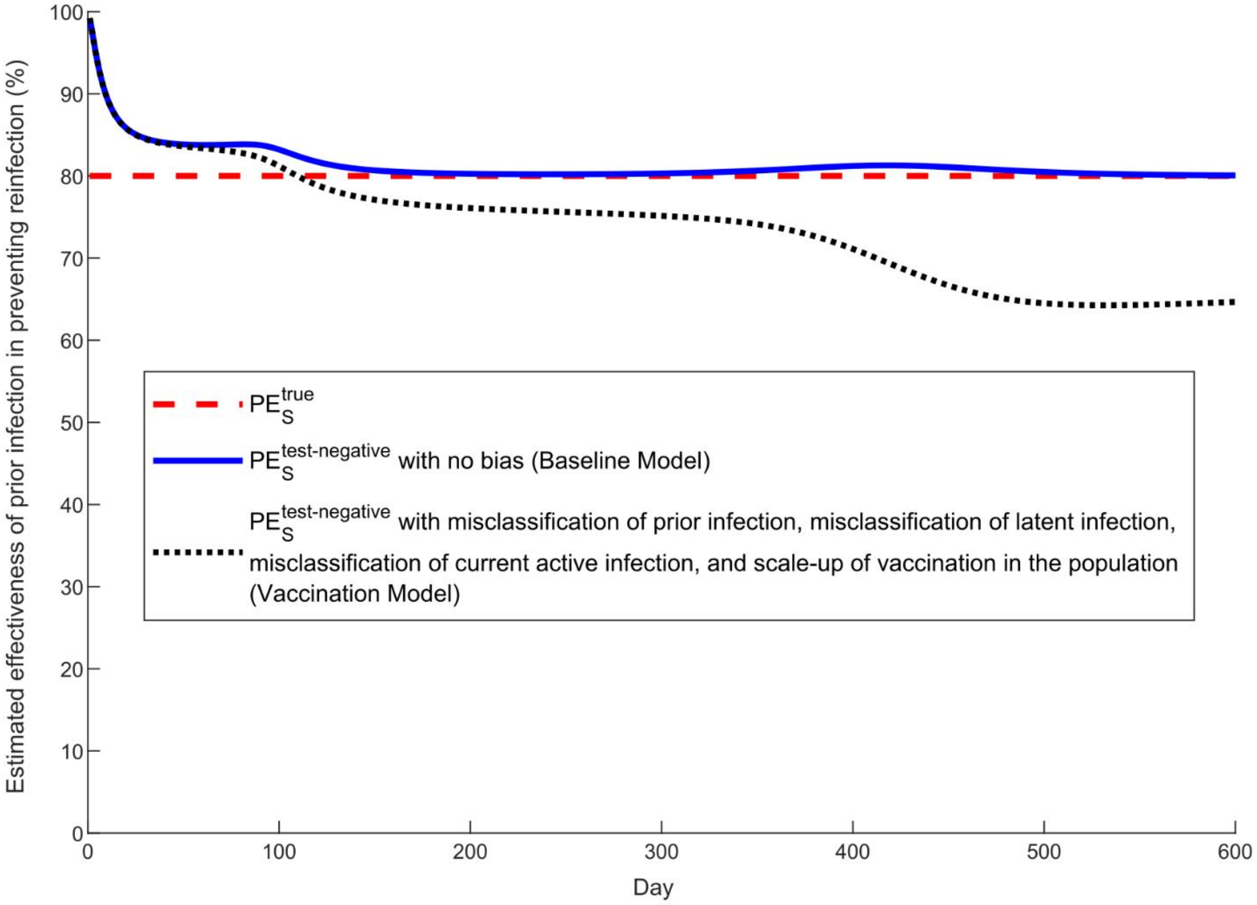

Figure S3. Sensitivity analysis. Impact of bias in estimating effectiveness of prior infection in preventing reinfection using the test-negative study design ($PE_S^{test-negative}$) applied to the data-fitted Qatar Model [1-4]. A) Impact of misclassification of prior infection. B) Impact of misclassification of latent infection. C) Impact of misclassification of current active infection.

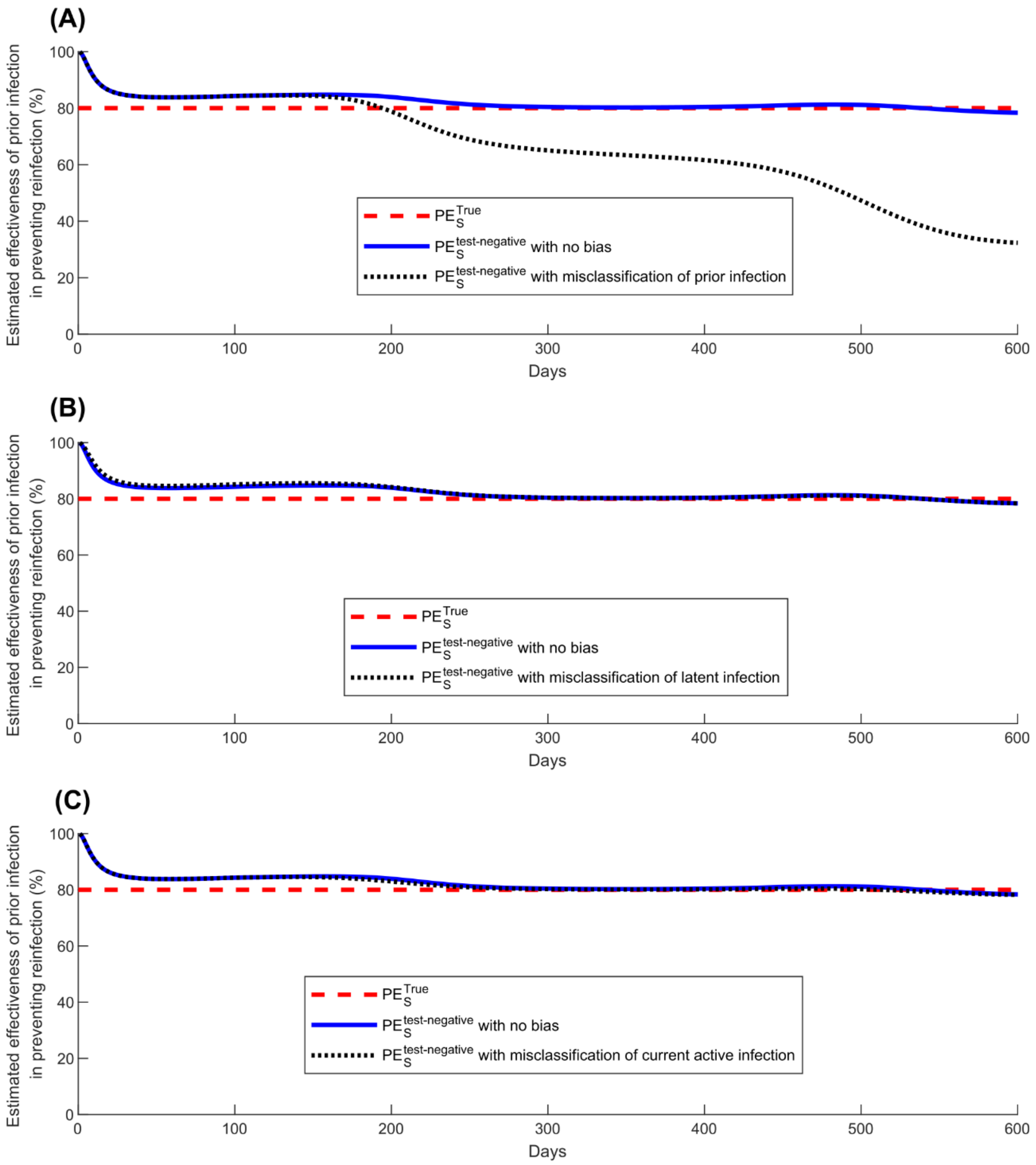

Figure S4. Sensitivity analysis. Investigation of the representativeness of the effectiveness of prior infection in preventing reinfection using the test-negative study design ($PE_S^{test-negative}$) of the true effectiveness (PE_S^{true}) over the full spectrum of possible PE_S^{true} values. $PE_S^{test-negative}$ is derived here at the infection endemic equilibrium. The figure shows also the linear trend fit of the relationship between PE_S^{true} and $PE_S^{test-negative}$.

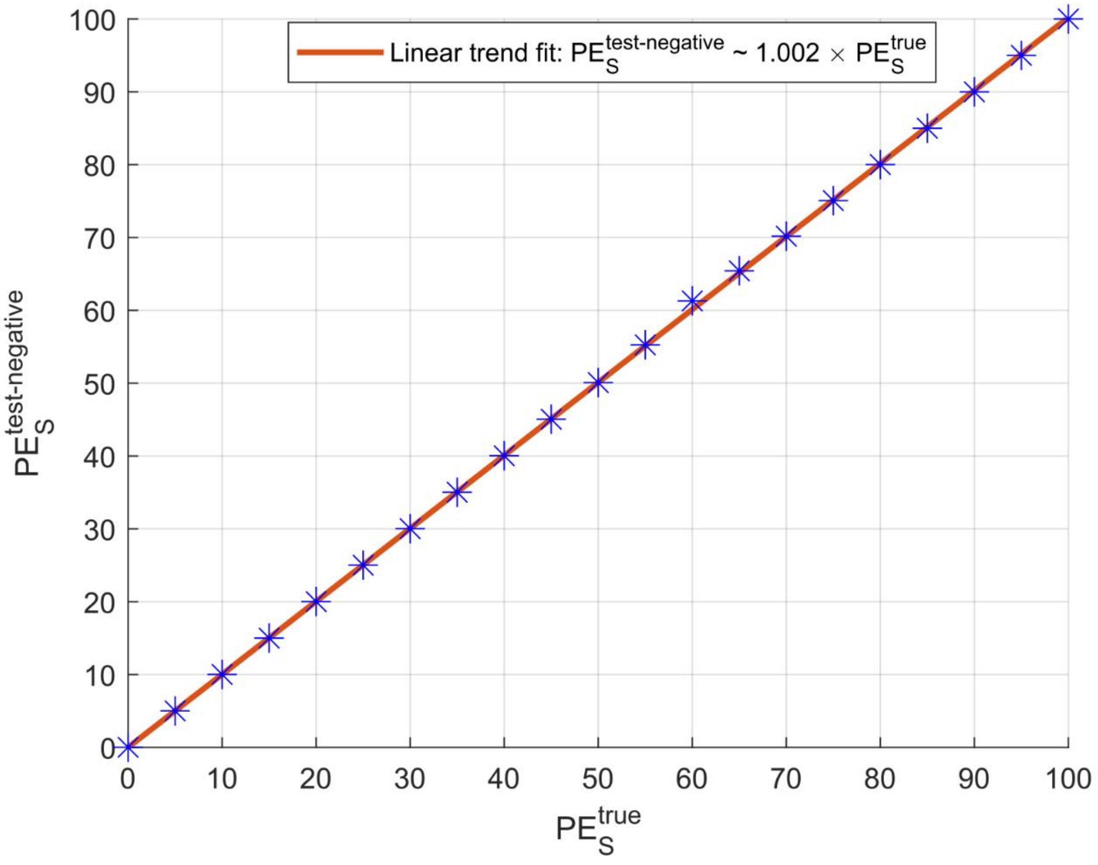

Figure S5. Sensitivity analysis. Impact of using instantaneous incidence instead of instantaneous prevalence in estimating effectiveness of prior infection in preventing reinfection using the test-negative study design ($PE_S^{test-negative}$).

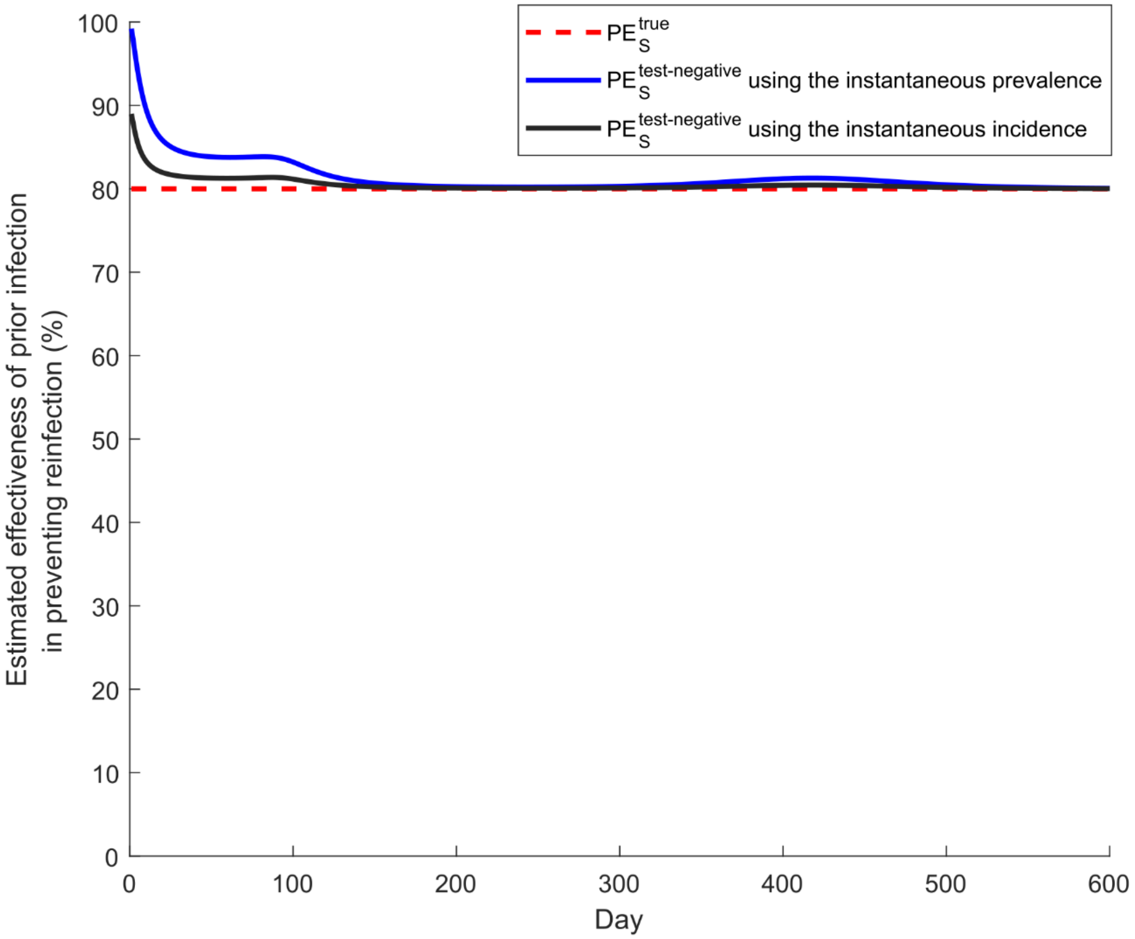

Figure S6. Sensitivity analysis. Impact on estimation of effectiveness of prior infection in preventing reinfection using the test-negative study design ($PE_S^{test-negative}$) if there is full misclassification bias of those latently infected. Here, none of those latently infected are being diagnosed; only those in acute infection are being diagnosed.

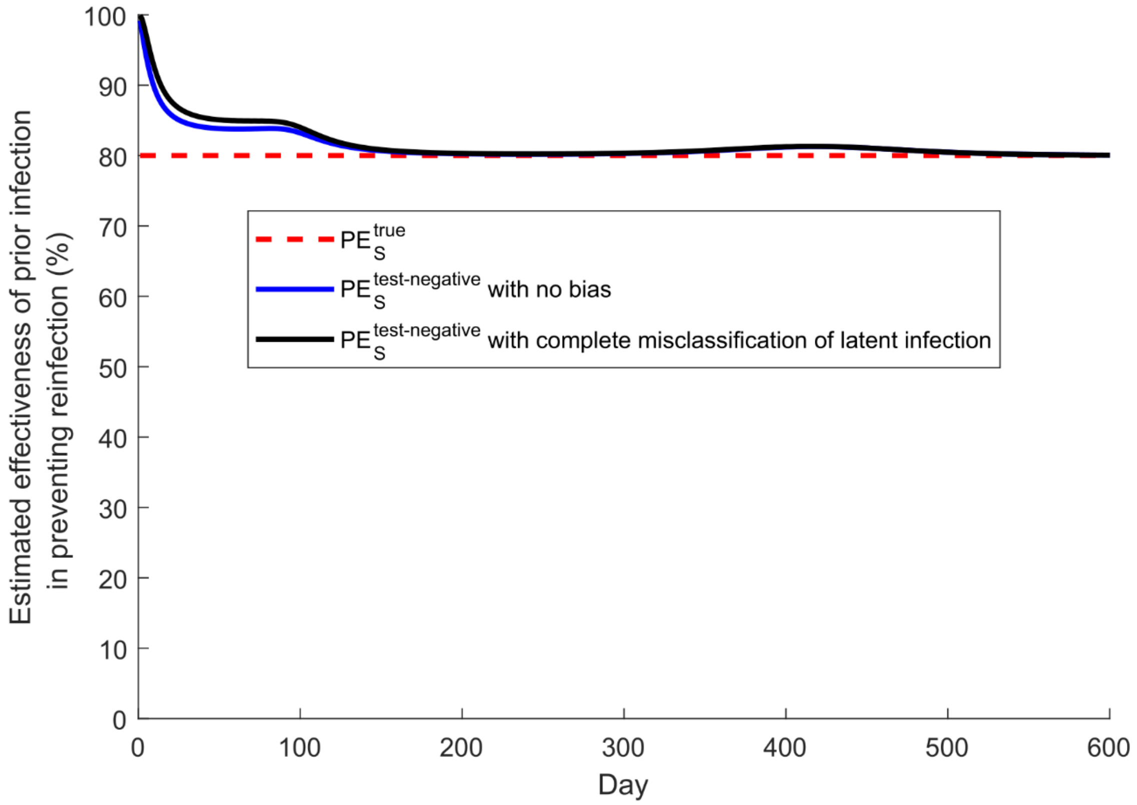

References

1 Ayoub HH, Chemaitelly H, Makhoul M, Al Kanaani Z, Al Kuwari E, Butt AA, et al. Epidemiological impact of prioritising SARS-CoV-2 vaccination by antibody status: mathematical modelling analyses. BMJ Innov. 2021;7:327-36.

2 Ayoub HH, Chemaitelly H, Seedat S, Makhoul M, Al Kanaani Z, Al Khal A, et al. Mathematical modeling of the SARS-CoV-2 epidemic in Qatar and its impact on the national response to COVID-19. J Glob Health. 2021;11:05005.

3 Bsat R, Chemaitelly H, Coyle P, Tang P, Hasan MR, Al Kanaani Z, et al. Characterizing the effective reproduction number during the COVID-19 pandemic: Insights from Qatar's experience. J Glob Health. 2022;12:05004.

4 Seedat S, Chemaitelly H, Ayoub HH, Makhoul M, Mumtaz GR, Al Kanaani Z, et al. SARS-CoV-2 infection hospitalization, severity, criticality, and fatality rates in Qatar. Sci Rep. 2021;11:18182.
